# Regional differences of testicular artery blood flow in post pubertal and pre-pubertal dogs

**DOI:** 10.1186/s12917-015-0363-3

**Published:** 2015-03-04

**Authors:** Mírley B de Souza, Claudia C Barbosa, Gary CW England, Antonio C Mota Filho, Carmen Vládia S Sousa, Gabriela G de Carvalho, Herlon Victor R Silva, José N Pinto, Jussiara CS Linhares, Lúcia DM Silva

**Affiliations:** Laboratory of Carnivore Reproduction, School of Veterinary Medicine, State University of Ceara (Universidade Estadual do Ceara), 1700, Doutor Silas Munguba Avenue, Fortaleza, CEP 60714-903 CE Brazil; School of Veterinary Medicine and Science, University of Nottingham, College Road, Sutton Bonington, Loughborough, Leicestershire, LE12 5RD UK; Vale do Jaguaribe Faculty, CE-040 Road, S/N, Km 38, Aracati, CE Brazil

**Keywords:** Dogs, Doppler, Ultrasonography, Testis

## Abstract

**Background:**

Measurement of testicular artery blood flow is used in several species to evaluate reproductive function and testicular and scrotal pathology. In dogs there are inconsistent reports about normal flow in post-pubertal dogs and no information concerning pre-pubertal dogs. The aim of this study was to describe regional differences in testicular artery blood flow in clinically normal post-pubertal and pre-pubertal dogs with no history of reproductive tract disease.

**Results:**

The post-pubertal dogs produced normal ejaculates throughout the study. In all dogs the three different regions of the artery were imaged and monophasic flow with an obvious systolic peak and flow throughout diastole was observed on every occasion. The highest peak systolic velocity (PSV) and end diastolic velocity (EDV) were measured within the distal supra-testicular artery and marginal artery whilst the lowest PSV and EDV were measured within the intra-testicular arteries. Flow measurements were not different between left and right testes and were consistent between dogs on different examination days. Calculated resistance index (RI) and pulsatility index (PI) were lowest in the intra-testicular arteries.

The pre-pubertal dogs had significantly smaller testes than the post-pubertal dogs (p < 0.05) and were unable to ejaculate during the study. The three different artery regions were imaged at every examination time point, and flow profiles had a similar appearance to those of the post-pubertal dogs. PSV, EDV, RI and PI showed a similar trend to the post-pubertal dogs in that values were lowest in the intra-testicular arteries. Notably, values of PSV, EDV, RI and PI were significantly lower (p < 0.05) in pre-pubertal dogs compared with post-pubertal dogs.

**Conclusions:**

This study demonstrated important regional and pubertal differences in testicular artery blood flow of dogs, and form the basis for establishing baseline reference values that may be employed for the purposes of clinical diagnosis.

## Background

Measurement of testicular artery blood flow using Doppler ultrasonography has been reported in a small number of studies in dogs [1-5]. An increase of testicular artery peak systolic velocity has been associated with testicular neoplasia [1], and more recently a relationship between testicular artery flow and semen quality was purported [6], although that study involved only five dogs.

In humans, the evaluation of testicular perfusion with Doppler ultrasound is well established [7,8], and changes in arterial flow have been described for the diagnosis of testicular and scrotal disease [9]. Such clinical diagnoses require comparison of the pathological testis with the contra-lateral testis and, especially for bilateral disease, an understanding of both positioning and patient factors that may influence the reference baseline [10]. Importantly, changes in flow characteristics have been observed in boys around the time of puberty [11] and it is clear that classification of Doppler flow characteristics according to age is important [12].

In dogs, the testicular artery has a complex appearance comprising, the convoluted supra-testicular region which is cranial to the testis, the marginal region which crosses the tunica albuginea and runs in the longitudinal plane to the caudal pole of the testis, and the intra-testicular branches which are directed towards the centre of the testis [13]. These authors also documented that proximally the supra-testicular region was loosely convoluted and that there were increased convolutions distally as the artery approached the cranial pole of the testis. Recently, the terms ‘cranial’ and ‘looping’ were used respectively to describe the proximal and distal portions of the supra-testicular artery; these regions were imaged in a comprehensive study along with the marginal artery and the intra-testicular branches [13]. Not all studies have used such clarity when describing the arterial regions, and it is possible that lack of consistency in anatomical description and recent improvements in the quality of ultrasound equipment may explain some of the conflicting results noted between studies; in particular whether blood velocities are higher in the supra-testicular or marginal artery and whether intra-testicular vessels can be reliably imaged [1-3].

It seems likely that in dogs, as in humans, there will be increasing attention paid to changes in artery flow as a diagnostic tool, and therefore baseline reference values are needed for both normal post-pubertal and normal pre-pubertal dogs. The aim of this study was therefore to establish the regional differences in testicular artery blood flow in post-pubertal and pre-pubertal dogs.

## Methods

### Experimental animals

This study was performed in the Laboratory of Carnivore Reproduction at the School of Veterinary Medicine, State University of Ceará and approved by the Animal Ethics Committee of the institution (protocol No. 12641034–8).

Ten post-pubertal dogs (2 Labrador, 4 Rottweiler, 4 German Shepherd) aged 2 to 4 years and weighing 33 to 42 kg, and 10 pre-pubertal dogs (4 Labrador, 3 Border Collie, 2 Golden Retriever, 1 Flat-Coated Retriever) aged 6 months and weighing 15 to 18 kg were used for the study. The dogs used in this study Belonged to São Lazaro Kennel 4° Cia de Choque/CPCÃES and the Estancia Kirst Kennel. A consent statement was signed by those responsible for the animals used in this research, explaining the purpose of the study and ensuring the welfare of animals.

Veterinary examination and complete blood count at the beginning of the study confirmed that all dogs were clinically normal and healthy. All dogs were fed a maintenance complete dry food with *ad libitum* water for the duration of the study.

### Breeding soundness evaluation

Dogs were selected from a larger group and only those with no previous reproductive disease and normal external genitalia were used [14]; all pre-pubertal dogs had not been bred and had small external genitalia but otherwise had a normal reproductive tract upon clinical examination.

Three ejaculates were collected from each of the post-pubertal dogs by digital manipulation at 7 day intervals during the study and the second fraction of the ejaculate was subjected to detailed examination. Attempts to collect semen from the pre-pubertal dogs were not successful. The second fraction was assessed immediately and volume was recorded. Subjective microscopic assessment of the percentage total sperm motility was made at ×400 magnification at room temperature. Sperm concentration was measured using a Neubauer chamber after dilution with formal-saline [15]. Membrane integrity was evaluated at ×400 magnification using the hypo-osmotic swelling test [16] and sperm morphology was evaluated at ×1000 magnification on Rose-Bengal stained slides [17], in each 200 sperm cells were evaluated.

### Ultrasonographic assessment

Three ultrasound examinations were performed on the right and left testis of each dog (after semen collection) with 7 days intervals, using a SonoAce PICO machine (Medison, Korea) with a linear array transducer with 5 to 9 MHz capability. Dogs were positioned in dorsal recumbency, acoustic gel was applied to the skin, and the transducer was positioned initially on the lateral surface of the testis. Longitudinal and transverse B-mode images were made (using the mediastinum as a reference point for measuring the testicular length and width) and testicular volume was calculated using the formula for an elipse; V = length × width × height × 0.5236 [18]. The appearance of the testicular parenchyma was recorded subjectively as heterogeneous or homogenous.

For the measurement of testicular artery flow in three separate regions, colour Doppler ultrasound was used with the transducer initially placed at the neck of the scrotum to identify the tortuous distal (looping) region of the supra-testicular artery (here termed distal supra-testicular artery), immediately cranial to the cranial pole of the testis. The transducer was then moved distally in the front (dorsal) plane to identify the marginal region in longitudinal section (here termed the marginal artery testicular artery), and the relatively straight intra-testicular arteries within the testicular parenchyma (here termed intra-testicular arteries). Within each region the colour gain was adjusted to reduce any excess colour noise and the pulsed Doppler gate was positioned within the lumen of the vessel. Three waves of a cardiac cycle were used to measure mean values for peak systolic velocity (PSV), end diastolic velocity (EDV), and these were used by the machine software to calculate resistance index (RI) and pulsatility index (PI). The sample gate ranged from 2.0 to 3.0 mm across all three regions and for two adult dogs was 5.0 mm for the supra-testicular artery only. The angle of insonation used was 0°. The same operator performed each examination.

The time taken to conduct the clinical, breeding soundness and ultrasound examination was recorded.

### Statistical analysis

Data were tested for normality (Shapiro-Wilk test) and homoscedasticity (Levene test). Data for semen quality, testicular volume and Doppler ultrasound measurements had a normal distribution; thus, analysis of variance of repeated measures was performed for the comparisons between weeks in each group evaluated, and the paired t test was used for the comparisons between the right and left testes. The t test was performed to assess the possible differences between the groups.

There were no significant differences (p > 0.05) between the left or right testes or between the examination time points, and so data were pooled as mean values for each dog. Pooled mean Doppler measurements between the different regions did not show homoscedasticity and so differences were assessed using the Friedman test. A significance level of p < 0.05 was used in all cases, and the results were expressed as the mean ± standard deviation.

## Results

The mean duration of the clinical, ultrasound and breeding soundness examinations was 45 minutes (range 30 – 70 minutes). There were no changes in the health or reproductive status of dogs during the study. Semen quality data for the post-pubertal dogs were normal [14] at all examination time points (Table 1).

**Table 1:** **Mean ± SD semen quality parameters of the ejaculate of 10 dogs (n = 30)**

**Parameters Evaluated**	**Mean ± SD**	**Range**
Volume (mL)	0.9 ± 0.6	0.2 – 1.5
Total motility (%)	95.0 ± 5.4	80 – 100
Sperm concentration (× 10^6^/mL)	1,218 ± 1,153	250 – 4950
Hypo-osmotic swelling test (% swollen)	89.5 ± 8.9	89 – 98
Morphologically normal spermatozoa (%)	86.7 ± 11.9	82 – 97

All testes were recorded as homogenous and the mediastinum testis was identified at each examination. Calculated testicular volume was significantly larger for post-pubertal (11.5 ± 2.1 cm^3^) compared with pre-pubertal dogs (4.3 ± 3.2 cm^3^) and did not change between weeks (data not shown).

Colour Doppler ultrasound allowed the identification of the testicular artery in both testes of all dogs in all regions at each examination. The distal supra-testicular artery had a tortuous pattern. Conversely, the marginal testicular artery had a more linear path and was observed in the longitudinal plane along the entire length of the testis. Tracing the marginal artery along the dorsal border of the testis allowed identification of a distinct curve around the caudal pole from which intra-testicular artery branches could be identified and which passed throughout the testicular parenchyma. Intra-testicular arteries were best identified using an imaging plane slightly oblique to the longitudinal plane, and here the intra-testicular arteries were found to have a linear path directed toward the mediastinum testis.

**Figure 1:**
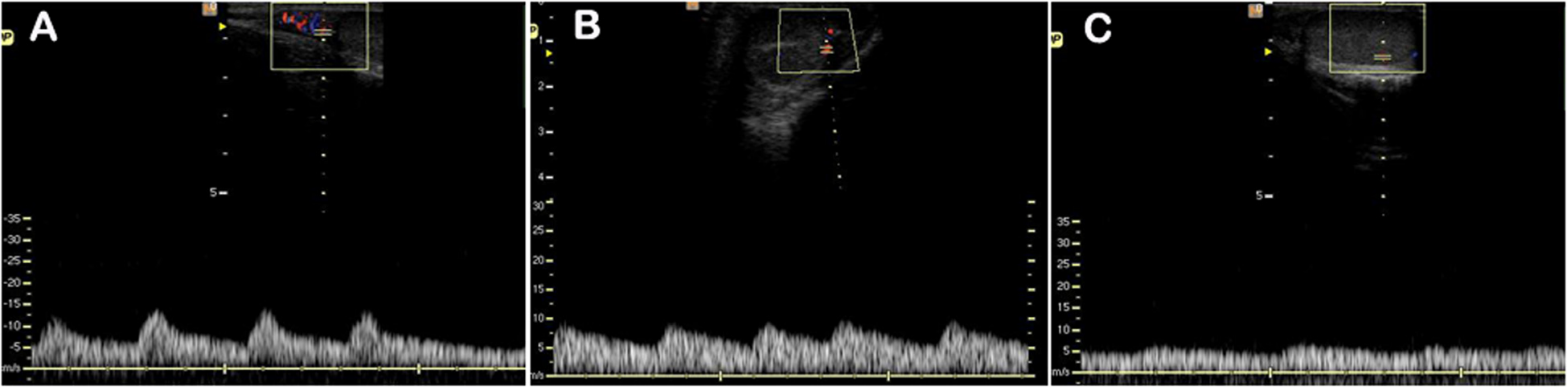
**Typical Doppler ultrasound waveforms from the testicular artery of post-pubertal dogs.** Images were recorded from **(A)** distal supra-testicular artery, **(B)** marginal artery, **(C)** intra-testicular artery.

**Figure 2:**
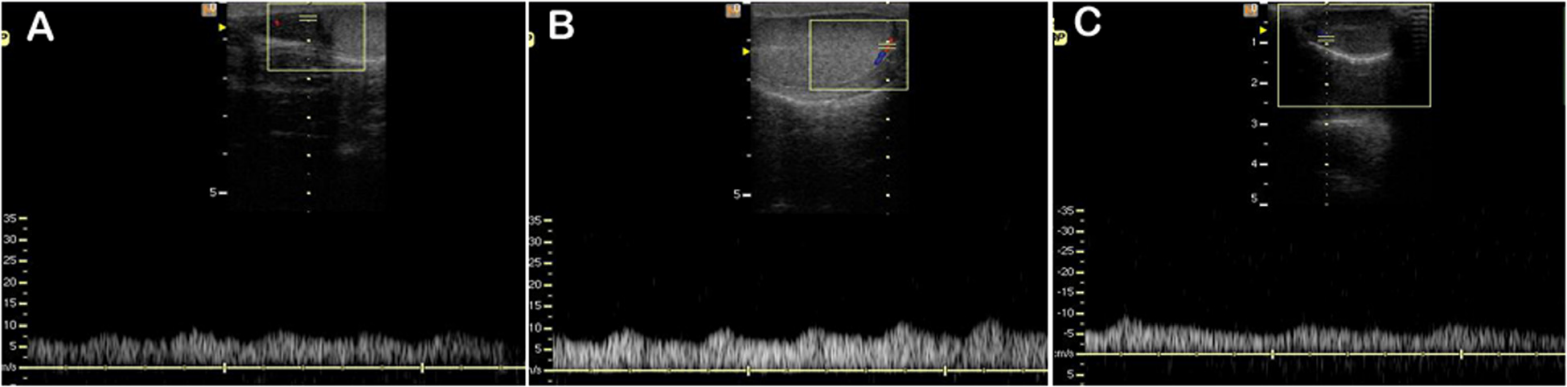
**Typical Doppler ultrasound waveforms from the testicular artery of pre-pubertal dogs.** Images were recorded from **(A)** distal supra-testicular artery, **(B)** marginal artery, **(C)** intra-testicular artery.

Doppler ultrasound showed that the testicular artery blood flow waveform was monophasic in each of the three separate regions, with systolic peaks, continuous diastolic flow and low vascular resistance. In the post-pubertal dogs the waveform recorded in the distal supra-testicular artery showed a more prominent systolic peak than in the other regions (Figure 1), and although similar this characteristic was not so pronounced in the pre-pubertal dogs (Figure 2).

There were no significant differences in Doppler measurements between the left or right testes or between the examination time points for either post-pubertal or pre-pubertal dogs.

For post-pubertal dogs there were regional differences in blood flow (Table 2). PSV in the distal supra-testicular artery and in the marginal testicular artery did not differ, but in both the PSV was higher than in the intra-testicular arteries. EDV showed a similar trend with significantly lower values in the intra-testicular arteries, but no differences between distal supra-testicular artery and the marginal testicular artery. These values resulted in differences in the calculated RI between all three regions with the highest RI in the distal supra-testicular artery and the lowest RI in the intra-testicular arteries. Similar trends were present with the PI where values were lowest in the intra-testicular arteries (Table 2).

**Table 2:** **Mean ± SD Doppler ultrasound parameters of the testicular arteries of post-pubertal and pre-pubertal dogs**

**Doppler parameters**	**Post-pubertal**			**Pre- pubertal**		
	**Region**			**Region**		
	**Distal supra-testicular artery**	**Marginal artery**	**Intra-testicular artery**	**Distal supra-testicular artery**	**Marginal artery**	**Intra-testicular artery**
PSV	13.34 ± 2.59^Aα^	11.27 ± 3.04^Aα^	6.87 ± 1.15^Aβ^	7.98 ± 1.86^Bα^	8.02 ± 2.03^Bα^	5.42 ± 0.84^Bβ^
EDV	6.71 ± 1.77^Aα^	6.85 ± 1.81^Aα^	4.73 ± 0.88^Aβ^	4.86 ± 1.24^Bα^	5.57 ± 1.58^Bα^	3.82 ± 0.69^Bβ^
RI	0.49 ± 0.12^Aα^	0.38 ± 0.08^Aβ^	0.32 ± 0.04^Aγ^	0.37 ± 0.11^Bα^	0.29 ± 0.09^Bαβ^	0.28 ± 0.05^Bβ^
PI	0.75 ± 0.29^Aα^	0.49 ± 0.15^Aβ^	0.39 ± 0.09^Aβ^	0.46 ± 0.14^Bα^	0.36 ± 0.13^Bβ^	0.33 ± 0.07^Bβ^

Different uppercase letters (AB) represent significant differences in the Doppler parameters of the respective region between the post-pubertal and pre-pubertal dogs (P <0.05). Different Greek letters (αβγ) represent significant differences in the Doppler parameters between regions of the Artery (P <0.05).

For the pre-pubertal dogs there were similar trends in regional blood flow to those seen in the post-pubertal dogs (Table 2). PSV and EDV were significantly lower in intra-testicular arteries compared with both the distal supra-testicular artery and the marginal testicular artery; the latter were not different to each other. Calculated RI was significantly higher in the distal supra-testicular artery, and the trend for higher PI within the distal supra-testicular artery was similar to that seen in post-pubertal dogs.

There were interesting differences between the post-pubertal and pre-pubertal dogs as values for PSV, EDV, RI and PI were significantly higher in post-pubertal compared with pre-pubertal dogs for all regions (Table 2).

## Discussion

The results of this study provide further insight into blood flow measured by Doppler ultrasound within the testicular artery of normal post-pubertal and pre-pubertal dogs and provide a useful reference baseline particularly with respect to differences according to region and pubertal status.

In the present study the three different regions of the testicular artery (distal supra-testicular artery, marginal artery, intra-testicular arteries) were reliably imaged in both testes of 10 post-pubertal and 10 pre-pubertal dogs on three occasions (120 individual examinations). The proximal region of the supra-testicular artery was not studied because it was not possible to ensure consistency of position between dogs. Interestingly, the intra-testicular arteries were most reliably imaged by following the route of the marginal artery and then within the testicular parenchyma using in plane slightly oblique to the longitudinal plane, similar to that noted in studies of men [7]. The anatomical location and the systolic peaks discernible above the diastolic flow were confirmatory that these vessels were the intra-testicular arteries.

The arterial profiles and measured parameters were found to be reproducible and did not change over the duration of the study. These results appear to offer improved reliability of imaging compared with one study which was able to identify the supra-testicular, marginal and intra-testicular arteries in only 70, 56 and 68% of cases respectively [2], and another one which found that intra-testicular arteries were not suitable for blood flow measurement [1]. It is most likely that these differences are accounted for by improved imaging equipment since those earlier studies were performed.

Doppler testicular artery blood flow profiles of post-pubertal dogs were monophasic with a defined systolic peak, continual diastolic flow and low vascular resistance. These results are similar to those that have been previously reported for the supra-testicular and marginal artery [1-4]. The blood flow profiles were consistent with arterial flow to and within other organs that require constant perfusion [19], and similar to those previously observed in human and dog testes [2,7]. Interestingly, one study [3] documented that the proximal supra-testicular artery (which was not examined in the present study) had a biphasic high resistance flow profile similar to that observed in stallions [20]. This observation likely relates to measurements being made in the artery closer to the abdominal aorta, and again reinforces the central message of our work that in order to establish reference baseline values it is imperative that anatomical location is precisely established.

Clearly absolute measurements of arterial blood velocity are difficult to compare between studies because of different equipment, patient and technical factors, however the PSV and EDV in the present study were similar in magnitude to those reported before [3] and showed a similar trend of decreasing PSV and EDV from the supra-testicular to the intra-testicular arteries. The relative changes of PSV and EDV resulted in a decreased RI and PI within the marginal artery and intra-testicular artery.

These results do not support the observations of another study [1] of higher PSV and EDV in the marginal artery compared with the distal supra-testicular artery. That report provided only a limited description of the methodology used for imaging the marginal artery, and this perhaps reinforces the view of the current authors about the need for careful position and patient description for the establishment of baseline reference values.

In the present study testicular artery blood flow profiles had a similar appearance in pre-pubertal dogs compared with the post-pubertal dogs although the systolic peaks were small. This trend has not been observed in studies in boys, where absent diastolic flow pre-puberty was reported in 67% of cases [11], however in that study the majority of pre-pubertal boys were only a few days old, and a later study showed that flow increased as puberty approached [21]. In the current study pre-pubertal dogs showed a trend for lower PSV and EDV within the intra-testicular artery as for post-pubertal dogs. Interestingly PSV and EDV were significantly lower in all regions for the pre-pubertal compared with post-pubertal dogs, and importantly differences in PSV and EDV were such that the RI and the PI were also lower in pre-pubertal dogs compared with post-pubertal dogs. Measurements of testicular artery blood flow before and after puberty are scarce. The aforementioned study in boys [11] purported to show higher RI before puberty, however data were not related to puberty rather an expected testicular volume at puberty, and RI was only measured within intra-testicular arteries where in 67% of cases no diastolic flow was detected, and the remaining cases were very young boys only a few days old. In the present study dogs immediately before and shortly after puberty were examined and significantly higher values of RI were demonstrated after puberty. Intuitively one might assume that RI would decrease at sexual maturity associated with vasodilation and increased blood flow, however, RI is a calculated value and is only a true representation of downstream vascular resistance when resistance is the sole variable [22]. It is likely that in these dogs there were differences in vessel length, wall thickness and elastic properties as have been reported in bulls [23], that would impact the calculated RI; clearly there were substantial differences in mean PSV and EDV between the post-pubertal and pre-pubertal group resulting in clear changes to the flow profile and calculated PI. It is also possible that comparisons between the post-pubertal and pre-pubertal group were compromised because different breeds were represented within the two groups, although the breeds were similar in body weight.

## Conclusions

These studies demonstrate important regional and pubertal differences in testicular artery blood flow of dogs, and form the basis for establishing baseline reference values that may be employed for the purposes of clinical diagnoses.
